# Do patients referred to emergency departments after being assessed in primary care differ from other ED patients? Retrospective analysis of a random sample from two German metropolitan EDs

**DOI:** 10.1186/s12245-023-00542-9

**Published:** 2023-09-26

**Authors:** Andreas Umgelter, Markus Faust, Slatomir Wenske, Katrin Umgelter, Roland M. Schmid, Georg Walter

**Affiliations:** 1 II. Medizinische Klinik, Klinikum Rechts Der Isar der Technischen Universität München, Munich, Germany; 2 Zentrale Notfallversorgung, Vivantes Humboldt Klinikum, 13509 Berlin, Germany; 3 Klinik Für Interdisziplinäre Intensivmedizin, Vivantes Humboldt Klinikum, Berlin, Germany; 4 Zentrale Notfallversorgung, Vivantes Klinikum Spandau, Berlin, Germany

**Keywords:** Primary health care, Emergency service, Hospital, Diagnostic imaging, Comorbidity, Quality of healthcare

## Abstract

**Background:**

To assess differences between patients referred to emergency departments by a primary care physician (PCP) and those presenting directly and the impact of referral on the likelihood of admission.

**Design of study:**

Retrospective cohort study.

**Setting:**

EDs of two nonacademic general hospitals in a German metropolitan region.

**Participants:**

Random sample of 1500 patients out of 80,845 presentations during the year 2019.

**Results:**

Age was 55.8 ± 22.9 years, and 51.4% was female. A total of 34.7% presented by emergency medical services (EMS), and 47.7% were walk-ins. One-hundred seventy-four (11.9%) patients were referred by PCPs. Referrals were older (62.4 ± 20.1 vs 55.0 ± 23.1 years, *p* < .001) and had a higher Charlson Comorbidity Index (CCI) (3 (1–5) vs 2 (0–4); *p* < .001). Referrals received more ultrasound examinations independently from their admission status (27.6% vs 15.7%; *p* < .001) and more CT and laboratory investigations. There were no differences in sex, Manchester Triage System (MTS) category, or pain-scale values. Referrals presented by EMS less often (9.2% vs 38.5%; *p* < .001). Admission rates were 62.6% in referrals and 37.1% in non-referrals (*p* < .001). Referral (*OR* 3.976 95% *CI*: 2.595–6.091), parenteral medication in ED (*OR* 2.674 (1.976–3.619)), higher MTS category (1.725 (1.421–2.093)), transport by EMS (1.623 (1.212–2.172)), abnormal vital parameters (1.367 (0.953–1.960)), higher CCI (1.268 (1.196–1.344)), and trauma (1.268 (1.196–1.344)) were positively associated with admission in multivariable analysis, whereas ultrasound in ED (0.450 (0.308–0.658)) and being a nursing home resident (0.444 (0.270–0.728)) were negatively associated.

**Conclusion:**

Referred patients were more often admitted. They received more laboratory investigations, ultrasound examinations, and computed tomographies. Difficult decisions regarding the necessity of admission requiring typical resources of EDs may be a reason for PCP referrals.

**Supplementary Information:**

The online version contains supplementary material available at 10.1186/s12245-023-00542-9.

## Background

Emergency departments are confronted with increasing numbers of patients commonly resulting in ED overcrowding, inefficient medicine, higher risk, and violations of patient dignity [1–3]. Many studies have examined subjective and objective reasons on the side of the patients that may lead to avoidable presentations to emergency departments [4–12].

Little is known about patients being sent to EDs by primary care. Studying a telephone triage-based service model in England, it has been found that one of five patients after PCP input was referred to an ED [13]. Recent data from Sweden showed that 13% of ED patients were sent by their PCP [14].

In Germany, three systems care for medical emergencies. In cases with no immediate danger to life, care is legal obligation of physicians in private practice licensed by the Association of Compulsory Health Insurance Physicians (ASHIPs). For off-hours service, these organize a 24/7 on-call duty and off-hours walk-in centers. Emergency medical services (EMS) care for “conditions that are life-threatening or cause concern that severe health impairment will ensue if not cared for immediately” as the Berlin law on EMS states [15] with similar regulations in the other German federal states. Hospital EDs take over these patients for further management. Patients may also directly approach EDs at their discretion without any fees or other disincentives. Legally, emergency care in hospital EDs is considered synonymous to in-patient hospital care. However, hospital EDs treat more than half of acute ambulatory cases in Germany [16] which, in 2019, amounted to 10.6 million urgent outpatient treatments [17]. Despite the seemingly strict formal separation between the ambulatory and hospital sectors, ED patients in Germany do not seem to differ much from those seen elsewhere, with up to 70% of presentations being discharged from emergency rooms after ambulatory care [18, 19]. Previous research indicated that EDs treat a higher proportion of injuries and use more technical resources than the off-hours services of the ASHIPs [20].

We believe that data on ED utilization may offer a vantage point for health system analysis. Hospital EDs as providers of last resort function as canary in the coal mine to detect healthcare needs that are not met in other areas of the healthcare or welfare systems [21].

There are only few publications concerning the interaction between the outpatients-sector run by the ASHIP and hospital EDs in Germany, and the available information is mainly based on aggregate insurance data [16, 17, 20]. In Germany, referrals from PCPs to general hospitals are not allowed for consultation but only with a prescription for admission. Anecdotal evidence and one previous German study in a university hospital context however indicate that these patients with a prescription for admission are often discharged from ED [19].

From an ED perspective, we set out to perform an exploratory study incorporating clinical and procedural data to better delineate differences between the group of patients sent by PCP and other patients presenting to the ED regarding their clinical profiles and diagnostic and therapeutic needs. We were also interested in the role of referral as a predictor of admission.

## Methods

All cases documented 2019 in the emergency departments of two metropolitan hospitals in Berlin, Germany, were extracted from the hospitals main-frame database. A random sample of 1500 patient visits was drawn from a total of 80,245 consecutive presentations. Manual analysis of digital charts was performed to include data on comorbidities according to the Charlson Comorbidity Index (CCI) [22], diagnoses, and resources used in the emergency room. Resources were each counted similar to the emergency severity index (ESI) [23–25], but in contrast to ESI, simple wound closure and application of casts and splints were counted as a resource to reflect work load and use of floor space under conditions of busy emergency rooms. The patient histories were screened for multiple presentations during the year preceding the current visit.

### Patient and public involvement

There was no patient or public involvement in this study.

### Statistical analysis

Sample size was set at 1500 cases. This number should allow for multivariable binomial regression analysis of events occurring in 10% of the sample size with about 15 predictors [26] minimizing the risk of overfitting of the model.

Patient data were inserted into a spreadsheet (Excel 16, Microsoft Corporation, Redmond, WA, USA) and analyzed in SPSS Statistics 26 (IBM, Armonk, NY, USA).

Analysis proceeded in three steps. First, the cohort was analyzed descriptively.

Proportions are given as percentages with their respective 95% confidence interval (CI). Normally distributed cardinal data are presented as mean ± standard deviation and not normally distributed cardinal and ordinal data as median (25th–75th percentile).

Unrelated groups were compared using chi-square test for nominal data and Mann–Whitney test for ordinal and cardinal data. *p*-values are given in the tables. Significance level was set to 0.05. To account for multiple testing, significance after conservative Bonferroni correction for 25 comparisons is indicated in the tables. Second, univariable and multivariable analyses were performed with admission as the dependent variable. To avoid overfitting [26], a limited number of predictors were selected for multivariable binomial logistic regression based on previous research, group differences between referred and unreferred patients, and univariable analysis and our research interest. Starting from the limited selection of variables, a stepwise backward exclusion of parameters with a significance level below 0.1 according to Wald was performed to test for significant influences on admission as a dependent variable. As a third step, the association of admission with referral and other predictors was retested in the subgroup of walk-ins, and the association of an ultrasound examination with referral was reviewed controlling for demographic, clinical, and other procedural parameters.

## Results

Thirty-two cases were inpatients transferred from other medical institutions, mainly two male correctional medical facilities. They were excluded from further analysis. Of the remaining 1468 patients (age 54.8 ± 22.9 years, 51.4% (48.8–54.0) female), 35.0% (32.6–37.4) had arrived by EMS, 12.2% (10.5–13.9) by patient transport services (PTS), 0.3% (0.0–0.6) by police, and 48.6% (46.0–51.2) were walk-ins. Manchester Triage System (MTS) categories were adjudicated as follows: red (0.6% (0.2–1.0)), orange (12.9% (11.2–14.6)), yellow (45.7% (43.2–48.2)), green (33.9% (31.5–36.3)), and blue (3.1% (2.2–4.0)). A total of 3.8% (2.8–4.8) of patients were directly seen by a physician and not triaged. In these, information on transport was also missing.

**Fig. 1:**
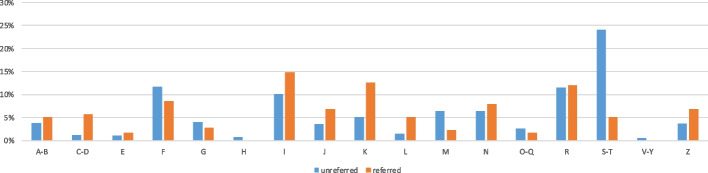
International Classification of Diseases, 10th revision, first digit and numbers of patients as percentages of the respective group total

Demographic, clinical, and procedural parameters of all patients are presented and compared according to referral status in Table 1, and diagnostic groups according to the 10th revision of the International Classification of Diseases are shown in Fig. 1. A Sankey diagram of patient flow is presented in Figure A of the data supplement.

**Table 1:** Comparison between patients who were or were not referred by a PCP

	All included patients (*n* = 1468)	Referral (*n* = 174)	No referral (*n* = 1294)	P (referral vs. non-referral) Level of significance after BF: .002
Age (in years) mean ± SD	55.8 ± 22.9	62.4 ± 20.1	55.0 ± 23.1	.00009*
Sex (female) (%)	51.4 (48.8–54.0)	52.9 (45.5–60.3)	51.2 (48.7–53.7)	0.685
Nursing home resident (%)	11.0 (9.4–12.6)	6.3 (2.7–9.9)	11.6 (10.0–13.2)	.037
Presentation during office hours (%)	64.2 (61.7–66.7)	77.6 (71.4–83.8)	62.4 (60.0–64.9)	.00009*
MTS triage level (median (25th–75th percentile)	3 (3–4)	3 (3–4)	3 (3–4)	0.383
Pain scale (VAS) (median (25th–75th percentile)	0 (0–0)	0 (0–0)	0 (0–0.25)	0.713
CCI (median (25th–75th percentile)	2 (0–4)	3 (1–5)	2 (0–4)	.0003*
Any laboratory investigations (%)	62.5 (60.0–65.0)	79.9 (73.9–85.9)	60.2 (57.7–62.7)	.00001*
ECG (%)	39.0 (36.5–41.5)	50.0 (42.6–57.4)	37.5 (35.1–40.0)	.001
Any imaging (%)	56.9 (54.4–59.4)	64.9 (57.8–72.0)	55.8 (53.3–58.3)	.022
• Plain films (%)	28.1 (25.8–30.4)	30.5 (23.7–37.3)	27.8 (25.5–30.1)	.465
• Ultrasound (%)	17.1 (15.2–19.0)	27.6 (21.0–34.2)	15.7 (13.8–17.6)	.00009*
• CT (%)	15.2 (15.2–19.0)	20.7 (14.7–26.7)	14.5 (12.7–16.3)	0.033
Simple procedure (%)	11.9 (10.2–13.6)	6.3 (2.7–9.9)	12.6 (10.9–14.3)	.016
Simple wound care (%)	7.0 (5.7–8.3)	1.1 (0.0–2.6)	7.8 (6.4–9.2)	.001
Parenteral medications (%)	27.2 (24.9–29.5)	25.3 (18.8–31.8)	27.5 (25.2–29.8)	0.536
> 1 resource (%)	66.1 (63.7–68.5)	74.7 (68.2–81.2)	64.9 (62.5–67.3)	.010
Resources (number) (median (25th–75th percentile)	2 (1–4)	3 (1–4)	2 (1–4)	0.006
Any abnormal vital parameters (%)	16.3 (14.4–18.2)	21.3 (15.2–27.4)	15.6 (13.7–17.5)	.058
Arrived with patient transport (%)	12.2 (10.5–13.9)	21.3 (15.2–27.4)	11.0 (9.4–12.6)	< .00001*
More than 3 visits to ED during past year (%)	13.4 (11.7–15.1)	5.2 (1.9–8.5)	14.5 (12.7–16.3)	.001
Arrived with EMS (%)	36.5 (34.0–39.0)	9.2 (4.9–13.5)	38.5 (36.0–41.0)	< .00001*
Trauma diagnosis (%)	17.9 (15.9–19.9)	4.6 (1.5–7.7)	19.7 (17.5–21.9)	< .00001*
Hospital admission (%)	40.1 (37.6–42.6)	62.6 (55.4–69.8)	37.1 (34.6–40.0)	< .00001*

*CCI* Charlson comorbidity index

^*^Significant after Bonferroni correction for 25 tests

Two-hundred forty-eight patients received ultrasound examinations, among these 220 abdominal examinations, 15 transthoracic cardiac ultrasound, and 23 examinations of the lower extremity veins. Laboratory investigations and ultrasound examinations were distinctly over-represented among referred patients.

The low number of referrals who were sent by EMS and the similar triage level and pain scale compared to unreferred patients indicate that most referred patients were not seen as under immediate threat in the a priori judgement of their referring PCP.

Seven-hundred eleven patients (age 48.3 ± 21.1; 49.2% (45.5–52.9) female) were walk-ins.

Under the assumption that PCP would send patients with severe acute conditions by EMS or at least PTS, we selected walk-ins as a group of referred patients, in whom other reasons than a grave medical condition requiring admission for referral to an ED may have been present, such as special diagnostic needs. To inquire why these patients may have been sent via the ED and not scheduled for direct admission to a ward, we compared these patients to walk-ins presenting without referral. The results are displayed in Table 2.

**Table 2:** Comparison of walk-ins who were or were not referred by a PCP

	All walk-ins (*n* = 711)	Referral (*n* = 117)	No referral (*n* = 594)	P (referral vs. non-referral) Level of significance after BF: .002
Age (in years) mean ± SD	48.3 ± 21.1	59.4 ± 19.5	46.2 ± 20.7	< .00001*
Sex (female) (%)	49.2 (45.5–52.9)	47.9 (38.8–57.0)	49.5 (45.5–53.5)	0.747
Nursing home resident (%)	0.8 (0.1–1.5)	0.0	1.0 (0.0–4.0)	0.257
Presentation during office hours (%)	66.1 (62.6–69.6)	81.2 (74.1–88.3)	63.1 (59.2–67.0)	.00016*
MTS triage level (median (25th–75th percentile)	3 (3–4)	3 (3–4)	3 (3–4)	0.939
Pain scale (VAS) (median (25th–75th percentile)	0 (0–0)	0 (0–1)	0 (0–0)	0.993
CCI (median (25th–75th percentile)	0 (0–3)	2 (0–4)	0 (0–2)	< .00001*
Any laboratory investigations (%)	53.7 (50.0–57.4)	76.1 (68.4–83.8)	49.3 (45.3–53.3)	< .00001*
ECG (%)	26.4 (23.3–29.6)	42.7 (33.7–51.7)	23.2 (19.8–26.6)	.000012
Any imaging (%)	47.7 (44.0–51.4)	58.1 (49.2–67.0)	45.6 (41.6–49.6)	.013
• Plain films (%)	20.7 (17.7–23.7)	17.9 (11.0–24.8)	21.2 (17.9–24.5)	0.426
• Ultrasound (%)	19.4 (16.5–22.3)	27.5 (19.4–35.6)	16.8 (13.8–19.8)	< .00001*
• CT (%)	9.1 (7.0–11.2)	21.4 (14.0–28.8)	6.7 (4.7–8.7)	< .00001*
Simple procedure (%)	9.3 (7.2–11.4)	4.3 (0.6–8.0)	10.3 (7.9–12.7)	.041
Simple wound care (%)	9.0 (6.9–11.1)	0.9 (0.0–2.6)	10.6 (8.1–13.1)	.0007*
Parenteral medications (%)	28.4 (25.1–31.7)	20.5 (13.2–27.8)	30.0 (26.3–33.7)	0.038
> 1 resource (%)	58.8 (55.2–62.4)	68.4 (55.2–62.4)	56.9 ()	0.021
Resources (number) (median (25th–75th percentile)	2 (1–3)	3 (1–4)	2 (1–3)	0.003
Any abnormal vital parameters (%)	14.2 (11.6–16.8)	17.1 (10.3–23.9)	13.6 (10.8–16.4)	0.327
More than 3 visits to ED during past year (%)	8.6 (6.5–10.7)	2.6 (0.0–5.5)	9.8 (7.4–12.2)	.011
Trauma diagnosis (%)	17.7 (14.9–20.5)	5.1 (1.1–9.1)	20.2 (17.0–23.4)	.0001*
Hospital admission (%)	27.1 (23.8–30.4)	53.0 (44.0–62.0)	22.1 (18.8–25.4)	< .00001*

*CCI* Charlson comorbidity index

^*^Significant after Bonferroni correction for 25 tests

A third of all patients who were referred with a prescription for in-patient treatment were discharged from the ED, representing a negative posteriori judgement by the ED regarding the need for admission. We present the 878 patients (age 50.2 ± 23.0; 53.5% (50.2–56.8) female) who were discharged from ED in Table 3, comparing those who had (*n* = 63) and those who had not been referred (*n* = 813).

**Table 3:** Comparison between referrals and non-referrals who were discharged from ED

	All patients discharged from ED (*n* = 878)	Referral (*n* = 65)	No referral (*n* = 813)	P (referral vs. non-referral) Level of significance after BF: .002
Age (in years)	50.2 ± 23.0	52.4 ± 19.9	50.3 ± 23.1	0.633
Sex (female) (%)	53.5 (50.2–56.8)	53.8 (41.7–65.9)	53.5 (50.1–56.9)	0.958
Nursing home resident (%)	10.1 (8.1–12.1)	1.5 (0–4.5)	10.8 (8.7–12.9)	.017
Presentation during office hours (%)	64.5 (61.3–67.7)	83.1 (74.0–92.2)	63.0 (59.7–66.3)	.001*
Triage level (median (25th–75th percentile)	3 (3–4)	4 (3–4)	3 (3–4)	0.205
Pain scale (VAS) (median (25th–75th percentile)	0 (0–0)	0 (0–1.5)	0 (0–0)	0.631
CCI (median (25th–75th percentile)	1 (0–3)	1 (0–3)	1 (0–3)	0.902
Any laboratory investigations %	44.0 (40.7–47.3)	49.2 (37.0–61.4)	43.5 (40.1–46.9)	0.374
ECG (%)	20.7 (18.0–23.4)	18.5 (9.1–27.9)	20.9 (18.1–23.7)	0.641
Imaging (%)	42.1 (38.8–45.4)	44.6 (32.5–56.7)	41.9 (38.5–45.3)	0.675
• Plain films (%)	19.3 (16.7–21.9)	15.4 (6.6–24.2)	19.7 (17.0–22.4)	0.402
• Ultrasound (%)	17.1 (14.6–19.6)	29.2 (18.1–40.3)	16.2 (13.7–18.7)	.007
• CT (%)	6.6 (5.0–8.2)	7.7 (1.2–14.2)	6.0 (4.4–7.6)	0.367
Simple procedure (%)	14.1 (11.8–16.4)	6.2 (0.3–12.1)	14.8 (12.4–17.2)	.055
Simple wound care (%)	10.3	3.1 (0.0–7.3)	10.8 (8.7–12.9)	.048
Parenteral medications (%)	17.5	6.2 (0.3–12.1)	18.4 (15.7–21.1)	.006
Resources: > 1	52.8	47.7 (35.6–59.8)	53.3 (49.9–56.7)	0.348
Resources (number) (median (25th–75th percentile)	2 (1–3)	1 (0–3)	2 (1–3)	0.166
Any abnormal vital parameters (%)	11.5 (9.4–13.6)	10.8 (3.3–18.3)	11.6 (9.4–13.8)	0.947
Arrived with EMS (%)	25.4 (22.5–28.3)	7.7 (1.2–14.2)	26.8 (23.8–29.8)	.0005*
More than 3 visits to ED during previous year (%)	12.2 (10.0–14.4)	4.6 (0.0–9.7)	12.8 (10.5–15.1)	.052
Trauma diagnosis (%)	22.3 (19.5–25.1)	7.7 (1.2–14.2)	23.5 (20.6–26.4)	.003

*CCI* Charlson comorbidity index

^*^Significant after Bonferroni correction for 25 tests

In univariable binary logistic regression analysis, age, referral, parenteral medication in the ED, higher urgency class in MTS, number of resources used, one or more abnormal vital parameters, higher CCI, and a trauma diagnosis were associated with admission (Table A, data supplement).

Due to missing data, only 1393 cases (94.9%) could be included in multivariable binary logistic regression analysis with admission as dependent variable. Age and pain scale were eliminated from the model without a significant decrease in model fit. The remaining independent predictors of hospital admission are displayed in Table 4. The model was statistically significant (*p* < 0.001), pseudo-R (Cox & Snell) was 0.340, and 77.2% of cases were correctly classified compared to 58.4% in the null model.

**Table 4:** Multivariable analysis of parameters associated with hospital admission in all patients

Parameter	Regression coefficient	*p*	Odds ratio	95% *CI*
Referral	1.380	< .001	3.976	2.595–6.091
Parenteral medication	0.984	< .001	2.674	1.976–3.619
MTS higher urgency per level	0.545	< .001	1.725	1.421–2.093
Transport by EMS	0.484	.001	1.623	1.212–2.172
Number of resources used	0.356	< .001	1.428	1.311–1.556
≥ 1 abnormal vital parameter	0.312	.090	1.367	0.953–1.960
CCI	0.237	< .001	1.268	1.196–1.344
Trauma	− 0.463	< .001	1.268	1.196–1.344
Ultrasound in ED	− 0.799	< .001	0.450	0.308–0.658
Nursing home resident	− 0.813	.001	0.444	0.270–0.728

An exploration of the written referral notes shows a wide variety of complaints, presentations, and suggestions. Suspicion of a surgical abdomen (*n* = 25, mostly suspicion of appendicitis or ileus) and dyspnea (*n* = 18, with a suspicion of pulmonary embolism in 4, acute heart failure in 3, or bronchial obstructive problems in 3) were the most common presentations.

The most common ICD groups of diagnoses of the 65 patients who were referred but discharged from ED were R10 (abdominal pain, *n* = 7), F32 (depressive episode, *n* = 3), I80 (thrombosis and thrombophlebitis, *n* = 3), and N39 (other diseases of the urinary tract, *n* = 3). The most common diagnoses of exclusion in these patients were acute coronary syndrome (*n* = 7), surgical abdomen (*n* = 6), bone fractures (*n* = 5), and urolithiasis (*n* = 3). Some referrals specifically asked for ultrasound investigations (*n* = 5) or other imaging which were the only specific interventions mentioned in the referral notes.

## Discussion

### Main findings

#### All patients

Our study finds a substantial proportion of patients who had been referred with a prescription for admission by their PCPs. One-third of referred patients and almost two-thirds of all other patients were discharged from the ED. Only a small minority of referred patients were transported by EMS. Most referred patients arrived on their own, presenting as walk-ins, which may indicate that they were not deemed to be under immediate threat by their referring PCP. A substantial number of ED resources was used in both referred and unreferred patients, and referred patients were almost double as likely to receive an ultrasound examination in the ED. They also were older, more often presented during office hours, had more comorbidities, and were less likely to have presented more than 3 times during the previous year. The proportion of nursing home residents was lower among referred patients, and they were rarely admitted. A large proportion of nursing home residents presented by patient transport for minor interventions such as wound dressing, diagnostics after falls, or changing urinary catheters. There were no differences in triage urgency, the number of abnormal vital parameters, or pain scale among referred and unreferred patients.

#### Walk-ins

Among walk-ins, who were overall younger and had less comorbidity than patients presenting by EMS or PTS, referred patients were older and had more comorbidities. Referred walk-ins had less often parenteral medication in the ED but used more diagnostic resources than walk-ins who were not referred.

Also, referred walk-ins were more likely to receive an ultrasound examination and more often underwent laboratory examinations and computed tomography than self-presenting walk-ins.

#### Patients discharged from ED

Among all patients discharged from ED, there were no relevant differences in age, comorbidity, vital parameters, or MTS triage level between referred and unreferred patients, but there was a tendency to more ultrasound examinations and parenteral medications in referred patients.

#### Predictors of admission

In multivariable analysis of all patients, the strongest predictor of hospital admission was referral by a PCP. Living in a nursing home and an ultrasound examination in the ED were associated with a lower likelihood of admission.

In the group of walk-ins, referral remained the strongest predictor of admission (Table B, data supplement), and ultrasound remained negatively associated with admission.

If referral as a walk-in is proof of a low estimated acuity or severity, other unaccounted medical parameters may have been the reason to refer via the ED. Higher age and more comorbidity in referred versus self-presenting walk-ins but not in referred versus self-presenting patients discharged from ED may hint to the possibility that comorbidity and age rather than acute problems were important reasons for admission. Also, the decision to discharge patients from ED may often have been dependent on diagnostic resources of the ED as suggested by the high proportion patients discharged from ED after ECG, imaging, or laboratory investigations. Bureaucratic and other barriers to scheduled admission may also have played a role.

#### Use of ED resources

Interestingly, in all analyzed groups of referred patients, ultrasound examinations were more common than in patients who were not referred. Referral was statistically associated with ultrasound examinations after controlling for trauma, MTS category, abnormal vital parameters, and admission (Table C, data supplement).

Laboratory investigations were also overrepresented among referred patients. In our EDs, these are often ordered in a standardized fashion according to presenting symptoms and may not reflect individual needs precisely. In referred patients, trauma presentations were rare, and the larger proportion of patients with laboratory investigations may correspond to a larger proportion of medical conditions and more comorbidity.

### Comparison with the literature

#### Patient itineraries and alternatives to ED visits

There are few studies describing patient itineraries from GP-type contacts to hospital EDs. Gries et al. found 7.7% among ED presentations to a German university hospital ED were referred patients, and 44.7% were self-referrals. OR for admission was 2.2 after referral by a PCP [19]. In that study, as in ours, trauma patients were rarely referred by a PCP. In a multicenter questionnaire study among walk-ins, 17% had been referred by their primary care physician and 8% by specialist referrals. We did not discriminate between these both groups of referring physicians because there were only few specialist referrals, and most of these were passing through referrals by PCP to EDs [5].

In times of widespread ED overcrowding, there is a focus on streaming patients with inappropriate presentations or PCP-substitutable conditions away from hospital EDs. Villareal et al. described a service model with telephone triage and involvement of PCP. Patients with face-to -face contact were more likely to be transferred to an ED than those with telephone contact only [13]. Recent studies from Sweden showed that more than half of patients presenting to a university level ED had been in contact with a PCP. A total of 81% stated they had been advised to visit the ED. Shorter symptom duration was a predictor of direct presentation to the ED [27]. Unfortunately, in our study, symptom duration was not documented. A nationwide study including all Swedish EDs found 13% of ED presentations being referred of which 27% were admitted [14]. The higher admission rate in our referred ED patients may be partially due to the fact that in Germany, referrals from PCP to EDs are always prescriptions for admission.

#### Barriers to boarding and use of ED resources

Barriers to planned boarding may have instigated PCPs to use EDs as access points to obtain hospital beds for patients who needed inhospital treatment but did not need ED interventions or acute care. The low triage level, and above all the fact, that most patients were not sent by EMS may hint in that direction. On the other hand, hospital resources were used to a large extent in these patients in the ED. A recent investigation of referrals from urgent care centers to hospital EDs in the USA found that advanced imaging studies in 40.7% (ultrasound 16%, *CT* 24.7%) and specialist consults were important reasons for ED referrals, whereas 55% of presentations did not receive any ED-specific treatment [28]. We believe, a likely explanation would be that in patients with acute symptoms, often the decision to admit requires extensive use of diagnostic means. This is the only study we identified that examined PCP — ED patient journeys with a focus on the use of ED-type resources. In this line, in our study, ultrasound was overrepresented in referrals and had a negative impact on the odds of admission. Computed tomography was more common in referrals than in non-referrals among walk-ins. From German insurance data, it has been hypothesized that increased availability of ultrasound, wound-care, and laboratory investigations in the ASHIP sector could improve out-patient emergency care [17] and reduce ED presentations. Our data regarding ultrasound would be consistent with this assumption.

#### Care during off-office hours and accessibility of hospital EDs

While a study from the Netherlands assumes that most patients during office hours visit their own PCP [29], we find a majority of walk-ins with low acuity visiting the ED without previous contact with their GP. Other studies have estimated that between 20 and 40% of patients in an ED were eligible for care by a GP [30]. Introduction of general practice cooperatives (GPC), where primary care cases can be seen during off-office hours, in Dutch EDs has resulted in a reduction of ED presentations by 30% and an increase of referrals by GPs or GPCs [31]. The rate of admissions from EDs also increased but was still comparatively low in relation to our figures. A study from Vienna also found a decrease of ED presentations after introduction of a primary care cooperative alongside the ED [32]. In Belgium, no change of ED case load has been found after installing a regional off-hours general practitioner cooperative [33]. Minderhout et al. surveyed patients’ motives to visit EDs instead of their GP and found that important issues were the subjective impression of severity of their condition but also the expectation that hospital infrastructure such as laboratory or radiological investigations might be needed [29]. In our cohort, the use of laboratory investigations and imaging was common even among discharged patients, suggesting that this perception of patients may be accurate. However, in referred patients, use of ultrasound and computed tomography was even higher, suggesting that this may have been the reason for referral, and that the decision of admission or discharge may have been dependent on this imaging.

#### Special populations

Referred patients in our cohort were older than direct ED presenters and were more co-morbid. Older patients have been found to be less likely to self-present with low-acuity conditions than younger patients [6]. This may be due to closer patient-physician relationships in older patients with increased medical needs [34, 35]. The proportion of frequent attenders in our cohort was similar to publications from the USA, Canada, and the UK [36–38]. Frequent users of the ED were rarely sent by their PCP, suggesting that in these cases the ED was rather used as a substitute for primary care than as an adjunct.

Nursing home residents were much less likely to present after contact with a PCP, and they were often cared for on an outpatient basis with little use of hospital resources. We suspect that some nursing home residents lack adequate access to primary care and are sent to emergency rooms often for simple medical assessment or simple measures as has been previously published [10, 39, 40]. In Germany, according to present regulations, this may be partly due to lacking reimbursement for patient transportation to ASHIP practices [41].

Wound care was underrepresented in referred patients. In Germany, PCP come from different professional backgrounds, but a substantial proportion of PCP do offer wound care and also suture lacerations. We were unable to obtain explicit data on this issue and did not identify any published evidence on this subject. It may be that most minor wound care is carried out by PCP, or that injured patients may primarily visit an ED.

### Limitations

This retrospective study has several limitations; whereas the selection of our random sample of a large number of consecutive patients during 1 year will not be biased, referrals and self-presentation to the two participating hospitals may have been influenced by regional factors and the specific services provided by both hospitals. It has been shown that PCP access varies dramatically throughout Germany [42]. Also, selection between EDs or PCP, respectively, will be influenced by socioeconomic factors that we could not account for. Our EDs serve two Berlin boroughs with a metropolitan and suburban setting and about 500,000 inhabitants with a below-average socio-economic status. Generalizations should be made with caution.

Sample size was planned based on rule-of-thumb estimates to allow for up to 15 predictors of events occurring in 10% of cases [26, 43]. This may be disputed as may be our selection of parameters included in multivariable analysis that was partly based on group differences, univariable analysis, known predictors of admission, and interest. Whereas we think risk of overfitting will be small, relevant parameters may have been overlooked. Results should be interpreted as preliminary and validated in other studies. Furthermore, our group comparisons are at risk of multiple testing [44]. This should be kept in mind while interpreting the results and planning further studies on the subject.

Except for 58 patients who were not triaged and who — due to the structure of our electronic admission protocols — also missed transport data, data completeness was good with the following caveats: CCI was calculated from the information contained in patients’ documents, but not collected prospectively from patients. It is probable that relevant information was missing, and it is possible that this underreporting disproportionately affected patients with limited access to healthcare and patients discharged from ED. Vital parameters were taken on an “as-needed” basis and were omitted in many patients who were judged to be stable, for example, those with minor injuries. These patients were counted as having no abnormal vital parameters.

Apart from referral notes, we had no information on the PCPs motives for referral. Further study of the expectations and motives behind informal modes of cooperation across sectoral barriers in the German health system may be of interest and should include PCPs.

### Clinical and policy implications

It appears that instead of a merely complementary operation of ASHIPs in the ambulatory sector, on the one hand, and hospital EDs in the in-patient sector, on the other hand — as codified in the strict regulatory separation between the sectors in Germany — in everyday life, PCP find more cooperative modes of care for their patients, thus overcoming sectoral borders and making use of ED infrastructure such as ultrasound or computed tomography for out-patient care. It is unclear if this is cost efficient. Better availability of ultrasound in primary care might help to reduce presentations to emergency departments. Provision of specialized nursing or home visits to nursing home residents may also reduce ED attendance.

### Supplementary Information

**Additional file 1: Supplementary tables: Table A.** Univariable nalysis with admission as dependent variable. **Table B.** Multivariable binary logistic regression: parameters associated with admission in the group of 711 walk-ins. 700 cases analyzed. The model is statistically significant (*p*<.001), pseudo-R (Cox&Snell): .269 and 79.0% of cases are correctly classified compared to 72.4% in the null model. **Table C.** Multivariable analysis of parameters associated with ultrasound in the ED – all patients. **Supplementary figure: Fig. A.** Sankey diagram of patient flow.

## Data Availability

Additional data are presented as supplement. We cannot share the original data due to data protection regulations allowing publication in aggregated form only.
