# Supplementary material for: Do patients referred to emergency departments after being assessed in primary care differ from other ED patients? Retrospective analysis of a random sample from two German metropolitan EDs
Source: Int J Emerg Med. 2023 Sep 26;16:64. doi: 10.1186/s12245-023-00542-9 (PMC10523768; doi:10.1186/s12245-023-00542-9)

Supplemental files:

Table A: Univariable Analysis with admission as dependent variable

| Parameter | Regression coefficient | p | odds ratio | 95% CI |
| --- | --- | --- | --- | --- |
| Age | .029 | <.001 | 1.029 | 1.024 – 1.035 |
| Pain Scale (VAS) | .022 | .443 | 1.022 | .967 – 1.080 |
| More than 3 Visits during past Year | .238 | .123 | 1.269 | .938 – 1.718 |
| Referral | 1.034 | <.001 | 2.812 | 2.027 – 3.901 |
| Parenteral medication | 1.463 | <.001 | 4.317 | 3.436 – 5.424 |
| MTS higher urgency per level | .914 | <.001 | 2.495 | 2.123 – 2.932 |
| Transport by EMS | 1.020 | <.001 | 2.774 | 2.218 – 3.469 |
| Number of resources used | .559 | <.001 | 1.750 | 1.635 – 1.873 |
| ≥ 1 abnormal vital parameter | -.844 | <.001 | .430 | .324 - .570 |
| CCI | .258 | <.001 | 1.294 | 1.240 – 1.351 |
| Trauma | .723 | <.001 | 2.061 | 1.536 – 2.764 |
| Ultrasound in ED | .008 | .953 | 1.008 | .764 – 1.332 |
| Nursing home resident | .213 | .206 | 1.238 | .889 – 1.723 |

Table B: Multivariable binary logistic regression: parameters associated with admission in the group of 711 walk-ins. 700 cases analyzed. The model is statistically significant (p<.001), pseudo-R (Cox&Snell): .269 and 79.0% of cases are correctly classified compared to 72.4% in the null model.

| Parameter | Regression coefficient | p | odds ratio | 95% CI |
| --- | --- | --- | --- | --- |
| Referral | 1.586 | <.001 | 4.884 | 2.920 – 8.169 |
| Parenteral medication | 1.326 | <.001 | 3.766 | 2.304 – 6.156 |
| MTS higher urgency per level | .478 | .002 | 1.613 | 1.191 – 2.184 |
| Number of resources used | .246 | .001 | 1.279 | 1.104 – 1.481 |
| ≥ 1 abnormal vital parameter | .476 | .092 | 1.609 | .026 – 2.797 |
| CCI | .282 | .001 | 1.326 | 1.201 – 1.463 |
| Trauma | -.583 | .081 | 5.558 | .290 – 1.075 |
| Ultrasound in ED | -.884 | .002 | .413 | .235 - .726 |
| Nursing home resident | -.715 | .547 | .472 | -041 – 5.432 |

Table C: Multivariable analysis of parameters associated with ultrasound in the ED – all patients.

| Parameter | Regression coefficient | p | odds ratio | 95% CI |
| --- | --- | --- | --- | --- |
| Referral | .519 | .009 | 1.681 | 1.140 – 2.478 |
| MTS higher urgency per level | .238 | .023 | 1.269 | 1.034 – 1.558 |
| Transport by EMS | .343 | .041 | .709 | .510 - .986 |
| Trauma | -1.154 | <.001 | .315 | .185 - .537 |
| Nursing home resident | -.461 | .084 | .631 | .374 – 1.064 |

Fig. A: Sankey diagram of patient flow


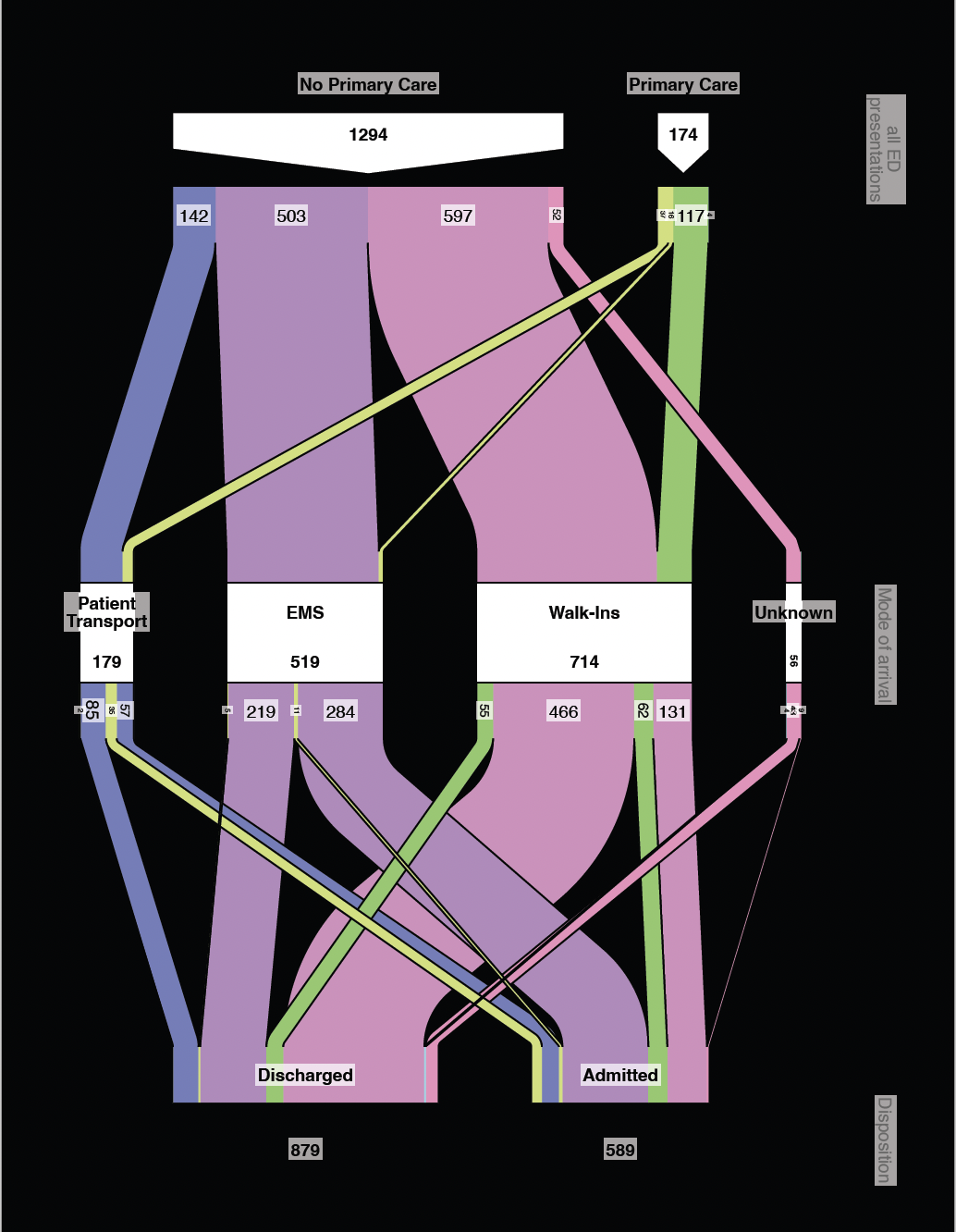

Supplement: Supplementary file 1 — Additional file 1: Supplementary tables: Table A. Univariable nalysis with admission as dependent variable. Table B. Multivariable binary logistic regression: parameters associated with admission in the group of 711 walk-ins. 700 cases analyzed. The model is statistically significant (p<.001), pseudo-R (Cox&Snell): .269 and 79.0% of cases are correctly classified compared to 72.4% in the null model. Table C. Multivariable analysis of parameters associated with ultrasound in the ED – all patients. Supplementary figure: Fig. A. Sankey diagram of patient flow. [file 12245_2023_542_MOESM1_ESM.docx]
